# Vascular Macrophages in Atherosclerosis

**DOI:** 10.1155/2019/4354786

**Published:** 2019-12-01

**Authors:** Hailin Xu, Jingxin Jiang, Wuzhen Chen, Wenlu Li, Zhigang Chen

**Affiliations:** ^1^Department of General Surgery, The First People's Hospital of Jiande, Hangzhou, China; ^2^Department of Surgical Oncology, Second Affiliated Hospital, Zhejiang University School of Medicine, Hangzhou, China; ^3^Key Laboratory of Tumor Microenvironment and Immune Therapy of Zhejiang Province, Hangzhou, China; ^4^Neuroprotection Research Laboratory, Massachusetts General Hospital, Harvard Medical School, Charlestown, MA, USA

## Abstract

Atherosclerosis is the main pathological basis for the occurrence of most cardiovascular diseases, the leading global health threat, and a great burden for society. It has been well established that atherosclerosis is not only a metabolic disorder but also a chronic, sterile, and maladaptive inflammatory process encompassing both innate and adaptive immunity. Macrophages, the major immune cell population in atherosclerotic lesions, have been shown to play critical roles in all stages of atherosclerosis, including the initiation and progression of advanced atherosclerosis. Macrophages have emerged as a novel potential target for antiatherosclerosis therapy. In addition, the macrophage phenotype is greatly influenced by microenvironmental stimuli in the plaques and presents complex heterogeneity. This article reviews the functions of macrophages in different stages of atherosclerosis, as well as the phenotypes and functions of macrophage subsets. New treatment strategies based on macrophage-related inflammation are also discussed.

## 1. Introduction

**Figure 1:**
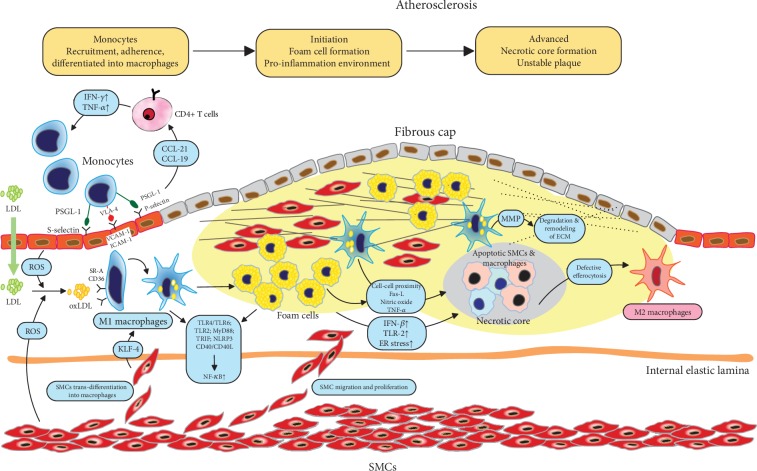
Roles of macrophages in different stages of atherosclerosis progression. Atherosclerosis is initiated by the subendothelial deposition of lipids. Circulating monocytes are recruited to the lesion site by adhering to activated endothelial cells (ECs) and entering the subendothelial cell space. Within the plaque, macrophages take up lipid deposit particles and transform into foam cells, forming early atherosclerotic lesions. Lesional macrophages further induce a cascade of inflammatory responses, promoting more lipoprotein retention, extracellular matrix (ECM) alteration, and sustained chronic inflammation. Oxidized LDL (oxLDL) further induces the necrosis of foam cells, which construct a necrotic core, leading to instability and rupture of advanced plaques. Abbreviations: CCL: chemokine ligand; ECM: extracellular matrix; ER: endoplasmic reticulum; Fas-L: Fas ligand; ICAM: intercellular adhesion molecule; IFN: interferon; IL: interleukin; KLF4: Kruppel-like factor 4; MMP: matrix metalloproteinase; NF-*κ*B: nuclear factor of kappa B; NLRP3: leucine-rich repeat pyrin domain containing 3; oxLDL: oxidized low-density lipoprotein; PSGL-1: P-selectin glycoprotein ligand-1; ROS: reactive oxygen species; SMC: smooth muscle cells; SR-A: type A scavenger receptor; TLR: toll-like receptor; TNF: tumor necrosis factor; TRIF: toll-like receptor domain-containing adaptor; VCAM: vascular cell adhesion molecule; VLA-4: very-late antigen 4.

Although much progress has been made in the diagnosis and treatment of cardiovascular disease (CVD) in recent years, CVD is still the leading cause of global morbidity and mortality [1]. The pathological cause of most CVD events, stroke, and peripheral arterial disease is atherosclerosis, thus motivating a number of researchers to study the pathophysiology of atherosclerosis over the past decades. Atherosclerosis is a focal vascular disease characterized by intimal thickening and plaque formation and mostly occurs at sites notably with endothelial cell injury and disturbed laminar flow [2]. Currently, it has been well established that atherosclerosis is both a component associated with metabolic disorder and a chronic inflammatory process in the arterial wall, which is induced initially by the subendothelial deposition of apolipoprotein B-containing lipoproteins (apoB-LPs) [3]. Macrophages, the major immune cell population in the arterial plaques, have been suggested to play a central role in the immune responses and progression of atherosclerosis (Figure 1) [2, 4]. Macrophages primarily originate from circulating monocytes and resident tissues. They are recruited to the lesion site by adhering to activated endothelial cells (ECs) and entering into the subendothelial cell space [5]. Then, macrophage proliferation becomes the predominant replenishment mechanism in advanced plaques [6]. Within the plaque, macrophages can take up lipid deposit particles and transform into foam cells, which is one of the hallmark events of the early atherosclerotic lesion [7]. These foam cells further induce a cascade of inflammatory responses that promote more lipoprotein retention, extracellular matrix (ECM) modification, and sustained chronic inflammation [8]. In addition, modified low-density lipoprotein (LDL), such as oxidized LDL (oxLDL), further induces the necrosis of foam cells, which can form a necrotic core, a typical feature of the instability of advanced plaques, leading to the rupture of plaques and further acute life-threatening clinical cardiovascular events [9]. Studies have concluded that increased lesional CD68^+^ macrophages are associated with a higher risk of CVD and stroke events, while presenting a weak relationship with stenosis [10, 11]. Therefore, clarifying the macrophage-dependent inflammatory processes in atherosclerosis progression and exploring macrophage-targeted strategies to reduce the residual risk of atherosclerotic CVD have become a hot research topic in recent years.

The macrophage phenotype is shaped greatly by microenvironment stimuli in the plaque, such as lipids, glucose, cytokines, and hemorrhage, and displays great plasticity [12]. Because complicated factors in the local milieu change with disease progression, macrophages are spatiotemporally heterogeneous. Traditionally, macrophages are classified into proinflammatory and anti-inflammatory phenotypes, which are well known as M1 and M2 phenotypes [13]. While in the plaque, this classification is reported to be an oversimplification of reality. In addition to M1 and M2, other macrophage subsets with distinct functions that do not resemble the M1/M2 transcriptomes and phenotypes have been reported [12, 14–17]. In addition, not only lesional macrophages but also circulating monocytes as well as their progenitor cells in the bone marrow are also stimulated by proatherogenic factors, such as cellular cholesterol content, and present great plasticity in genetic and epigenetic characteristics [18]. Owing to these functional complexities, although amply documented preclinical models are reported, few clinical trials have been developed to therapeutically target macrophages.

In this review, we will focus on the recent evidence on macrophage pathophysiology, presenting an overall view of the critical role of macrophages in different stages of atherosclerosis and their functional diversity. Moreover, we will review and discuss the major clinical strategies to modify macrophage-dependent chronic inflammation processes in plaques. Finally, we will highlight macrophages as a potential therapeutic target in atherosclerosis.

## 2. Origin of the Plaque Macrophage

Macrophages are considered to mainly originate from circulating monocytes, which are derived from the bone marrow [19] or spleen [20], which is widely known as the mononuclear phagocyte system (MPS). Monocytes in the circulation are recruited to the specific tissue site by various inducers such as tissue injury, pathogens, and proinflammatory cytokines and chemokines. Based on thymidine pulse-labeling animal models, van Furth et al. proposed that macrophage population replenishment was mainly dependent on monocyte recruitment [21]. Recently, this conclusion was challenged by the results from genetic fate mapping studies (tracing cell lineages) of tissue-resident macrophages, such as Langerhans cells, lung alveolar macrophages, Kupffer cells, and microglia [22–25]. These tissue-resident macrophages are established during fetal development and mostly maintain and replenish themselves by proliferation [24].

Recently, lineage fate mapping studies of vascular smooth muscle cells (VSMCs) in murine models demonstrated that VSMC subsets with highly proliferative plasticity can also transdifferentiate into macrophage foam cells [26]. This is in accordance with the earlier findings that lesional foam cells coexpressed VSMC markers [27, 28] and activation of the transcription factor Kruppel-like factor 4 (KLF4) may be the critical mechanism [29]. However, *in vivo* studies found that these VSMC-derived macrophage-like cells are different in transcriptional profiles and functions compared to classical macrophage [29, 30], such as in phagocytosis or efferocytosis [31].

In addition to exogenous replenishment, the progression of advanced atherosclerotic lesions is mainly dependent on local cell proliferation, which is involved in focal intimal thickening of the human aorta and further contributes to the progression of atherosclerosis [6, 32].

## 3. Macrophages in the Initiation of Atherosclerosis

### 3.1. Monocyte-Endothelial Cell Adhesion

Monocyte-endothelial cell adhesion plays a key role in the initiation of atherosclerosis. Complicated signaling pathways are involved in this process, and among them, the most notable pathway is the interaction between P-selectin glycoprotein ligand-1 (PSGL-1) and selectins [33]. Activated lesional ECs express P- and E-selectin [34, 35]. Selectins bind to the properly glycosylated PSGL-1, their predominant ligand that is expressed on monocytes and leukocytes [34, 35]. Selectin-PSGL-1-mediated interactions promote the capture of both monocytes and leukocytes onto the endothelium, activate integrins, and induce monocyte activation [36, 37]. In addition to the adhesion functions, PSGL-1 interacts with chemokine ligand (CCL) 21 or CCL19 and efficiently attracts activated CD4^+^ T cells to the vulnerable plaques [38, 39]. These CD4^+^ T cells produce interferon- (IFN-) *γ* and tumor necrosis factor- (TNF-) *α* and contribute to the proinflammatory environment. In accordance with these findings, knockout of PSGL-1 in *ApoE*^−/−^ mouse models showed less monocyte and leukocyte infiltration in atherosclerotic lesions and protection against atherosclerosis [40, 41]. Research based on double knockout mouse models including *P-selectin*^−/−^*ApoE*^−/−^ and *E-selectin*^−/−^*ApoE*^−/−^ mice also indicated decreased atherosclerosis formation [42, 43].

The binding of selectins to their ligands allows monocytes in circulation to be tethered and roll along the endothelium, and the subsequent ligation of monocyte integrins with vascular cell adhesion molecule 1 (VCAM1) or intercellular adhesion molecule 1 (ICAM1) on the ECs constructs a firm adhesion between monocytes or lymphocytes and ECs [33]. The most relevant integrin is very-late antigen 4 (VLA-4), also known as *α*4*β*1 integrin [44], and is widely expressed on monocytes and lymphocytes and can bind with VCAM1, which is overexpressed on activated ECs [45]. When mouse models lacking one of the two VCAM1 ligand binding sites were double hit by LDL receptor knockout (*LDLR*^−/−^), the mice developed reduced atherosclerotic lesions under a proatherogenic diet [46]. Utilizing *in vitro* studies, researchers found that after blockade of VLA-4 or VCAM1 by monoclonal antibodies, mononuclear cells rolled faster along the carotid arteries isolated from *ApoE*^−/−^ mice than those in the control, and monocyte accumulation onto the endothelium was reduced by over 70% [47, 48]. Activated platelets on the inflamed endothelium also contribute to monocyte-endothelial interactions via augmentation of adhesion selection expression and secretion of proinflammatory chemokines such as CCL5 [49]. In addition, C-C chemokine receptor type (CCR) 2, CCR5, and CX3C chemokine receptor 1 (CX3CR1) signals are indicated to contribute to the migration of monocytes into arterial walls [50–52]. In the *ApoE*^−/−^ mouse model, inhibition of three pathways, including CCL2, CX3CR1, and CCR5, almost abrogates macrophage accumulation and atherosclerosis (90% reduction), which is significantly more than the 28%, 36%, or 48% reduction in *ApoE^−/−^ CCL2^−/−^*, *ApoE^−/−^ CX3CR1^−/−^*, and *ApoE^−/−^ CCL2^−/−^ CX3CR1^−/−^* murine models, respectively [50]. Moreover, IFN-*β* signaling also enhances macrophage-endothelial cell adhesion and promotes immune cell infiltration to atherosclerosis-prone sites in mice, leading to the acceleration of lesion formation [53].

### 3.2. Macrophages and Foam Cells

After adhering to the ECs, monocytes penetrate through ECs into the subendothelial space and stay there because of their decreased migration ability, hindering the resolution of inflammation. Driven by prodifferentiation factors such as macrophage colony-stimulating factor (M-CSF), monocytes give rise to macrophage- or dendritic cell- (DC-) like phenotypes. These cells actively participate in scavenging lipoprotein particles and turn into foam cells, which present cytoplasmic and membrane-bound droplets, resulting in more accumulation of oxLDL in the subendothelial space [54, 55]. Several mechanisms have been proposed for this uptake process. Scavenger receptors expressed on the macrophages, especially the type A scavenger receptor (SR-A) and a member of the type B family, CD36, have been reported in early studies to be the main markers on lesional macrophages that transform into foam cells [8, 56]. Blocking SR-A inhibits the uptake of lipids and formation of foam cells, further prohibiting the local proliferation of macrophages in the lesion [6]. However, in triple knockout *Apoe^−/−^ CD36^−/−^ Msr1^−/−^* mouse models, no decrease was observed in the foam cell transformation compared with that of *Apoe^−/−^* mice, indicating that more mechanisms controlling this process remain to be clarified [57]. Recently, more novel scavenger receptors have been identified, such as LDL receptor-related protein 1 (LRP1) and lectin-like oxLDL receptor 1 (LOX1), which also contribute to lipid uptake [58, 59]. Blocking LRP1 in lesional macrophages has been proven to reduce the accumulation of cholesterol in macrophages [59]. In contrast, liver X receptor (LXR), which is activated by oxLDL, promotes the outflow of cholesterol and reduces the expression of proinflammatory factors in macrophages, thus exerting a favorable effect on atherosclerosis [60]. In addition to oxLDL, Kruth et al. found that foam cell transformation could also take place via intake of native LDL independent of receptors [61]. This process is called fluid-phase endocytosis and relies on the activation of phorbol 12-myristate 13-acetate (PMA), the activator of protein kinase C (PKC).

### 3.3. Macrophages and Proinflammatory Cytokines

Foam cells secrete abundant proinflammatory cytokines and in turn promote the accumulation and proliferation of circulating monocytes. Toll-like receptors (TLRs) have been proven to play a critical role in inflammatory signaling cascades. TLRs are essential pattern recognition receptors that mediate innate immune responses during invading pathogen invasion, such as viral and bacterial infection [62]. Phospholipid-CD36 binding on the lesional macrophages induces TLR4/TLR6 heterodimer formation, followed by activating downstream molecules, including myeloid differentiation factor 88 (MyD88), interleukin (IL)-1, toll-like receptor domain-containing adaptor (TRIF), and nuclear factor of kappa B (NF-*κ*B) [63]. In accordance with these reports, studies based on the mouse model have demonstrated that gene deletion of TLR2, TLR4, or MyD88 results in a reduction in atherosclerosis [64–66]. Endothelial-targeted blocking of NF-*κ*B signals in the *Apoe*^−/−^ mouse model resulted in a reduction in recruitment of macrophages to lesions [67]. Macrophage inflammasome signaling also plays a role in atherosclerosis. Crystalline cholesterol induces IL-1 family cytokines in macrophages by stimulating the caspase-1-activated nucleotide-binding domain and leucine-rich repeat pyrin domain containing 3 (NLRP3) inflammasome [68]. The NLRP3 inflammasome, as the most well-known inflammasome, is essential for necrotic core formation in advanced atherogenesis, and its silencing protects the stabilization of atherosclerotic plaques [69]. Except for B cells, ECs, SMCs, and platelets also express CD40 when induced by proinflammatory stimuli, such as IL-1, IL-3, IL-4, TNF-*α*, and IFN-*γ* [70]. Gene-targeting studies utilizing murine knockout models have established that CD40L participates in lesion progression and thrombosis [71]. *In vitro* studies indicate that ligation of CD40/CD40L stimulates proinflammatory cytokines and cell adhesion factors in vascular endothelial cells [72].

## 4. Macrophages in Advanced Atherosclerosis

### 4.1. Macrophages and Fibrous Caps

Stable plaques with intact fibrous caps rarely cause detrimental symptoms owing to the preservation of the arterial lumen, which relies on matrix metalloproteinase- (MMP-) mediated vascular remodeling [73, 74]. A plaque becomes unstable when the fibrous cap becomes thin and a necrotic core arises, followed by its breakdown from the endothelia and further acute, occlusive lumenal thrombosis, leading to thromboembolic events such as heart attack or stroke and high mortality [9].

Lesional macrophages promote the apoptosis of smooth muscle cells (SMCs) in the plaque in several ways, including cell-cell proximity [75] and activation of multiple cytotoxic signals including Fas-L, nitric oxide (NO), and TNF-*α* [76, 77], thus predisposing the plaque to rupture. Collagen synthesis by intimal SMCs is also reduced due to decreased macrophage-derived TGF-*β* [78, 79]. In addition, lesion macrophages promote extracellular matrix (ECM) remodeling by producing MMPs, especially MMP-2 and MMP-9, which can induce ECM protein degradation, thinning of the fibrous cap, and the formation of rupture-prone plaques [80]. Notably, different MMP members may play divergent roles during the atherosclerosis process, and MMPs present a dual role in this progression by promoting the migration and proliferation of vascular smooth muscle (VSMC) in the early stage while accelerating plaque instability by matrix destruction in advanced atherosclerosis [81]. For example, Gough et al. found that overexpression of an activated MMP-9 mutant (MMP-9 G100L) contributed to fibrous cap disruption, thrombus formation, plaque rupture, and mouse mortality in an *Apoe^−/−^* mouse model [82]. However, Johnson et al. observed a contradictory unfavorable effect on plaque size and stability when MMP-9 was knocked out in an *Apoe^−/−^* mouse model [83]. Therefore, more studies of MMP knockout or overexpression are needed to resolve the dispute that most likely results from differences in sites and stages of plaque development, assessment of plaque instability, dietary treatment, and mouse model strains. In addition, limitations such as utilization of indirect evidence as an endpoint for plaque rupture and a lack of acute lumenal thrombosis similar to that in human lesions in previous mouse model studies also restrain the application of these findings for clinical trials.

### 4.2. Macrophages and Necrotic Cores

The second feature of advanced plaques is the formation of necrotic cores. Generally, the necrotic core of a plaque is a hallmark of plaque vulnerability and contributes to nonresolving inflammation, thrombosis, fibrous cap breakdown, and plaque rupture [9]. The necrotic core is mainly composed of apoptotic lesional macrophages and defective phagocytic clearance [84]. A number of signals participate in necrotic core formation, including growth factor deprivation, oxidative stress, and death receptor activation by ligands [2]. In *Apoe^−/−^* mice, IFN-*β* not only induces the recruitment of macrophages to the lesion but also contributes to cell apoptosis and further necrotic core formation [53]. Similarly, oxLDL-CD36 complex-triggered TLR2-dependent signaling promotes the initial proinflammatory environment and further induces apoptosis of endoplasmic reticulum- (ER-) stressed macrophages [56].

ER stress, primarily the unfolded protein response (UPR), is a novel apoptotic mechanism discovered in recent years and has been proven to play critical roles in proatherosclerotic inflammation, necrotic core formation, and atherosclerosis plaque progression [85]. Factors associated with cardiovascular diseases are reported to be potent inducers of prolonged ER stress, including insulin resistance and obesity [86–88]. The expression of the UPR effector, C/EBP homologous protein (CHOP), shows a strong correlation with the progression of human coronary artery lesions [89], and knockdown of CHOP expression *in vitro* decreases ER stress-dependent cell death [90, 91]. In addition, considerable studies have highlighted that ER stress is involved in the inflammation processes within the lesion through manipulating a variety of regulators, such as suppressing NF-*κ*B signaling and activating activator protein-1 (AP-1), Jun amino-terminal kinases (JNK), spliced X-box binding protein 1 (XBP1), and reactive oxygen species (ROS) [92–95]. In addition, prolonged ER stress and abnormally activated UPR are also related to overactive autophagy, causing SMC and EC death and finally leading to a thinner fibrous cap [96]. *In vitro* studies indicated that nitric oxide (NO) donors, such as Molsidomine, spermine NONOate, or S-nitroso-N-acetylpenicillamine (SNAP), can preferentially eliminate macrophages in an ER stress-dependent manner and favor the stability of plaques [95].

In advanced lesions, macrophage apoptosis is followed by defective efferocytosis, which is the key driver for necrotic core formation [97]. Compared with the normal tonsil tissue in which each of the apoptotic cells was associated with a phagocyte, there were many free apoptotic cells in the advanced lesion [97]. Several mechanisms are proposed to contribute to this efferocytosis failure, including changes in the phenotypes of plaque cells that express markers such as CD47 and are poorly internalized by lesional efferocytes [98], reduced “eat me” signal calreticulin on the apoptotic cells [99], competition between the apoptotic cells and oxLDLs in binding to efferocytosis receptors [100], oxidative stress-induced efferocyte death [101], and the deficient expression and function of efferocytosis receptors as well as their bridging molecules such as MerTK-Gas6 [102]. Blocking CD47 and protecting MerTK on apoptotic macrophages to enhance efficient efferocytosis are potential strategies to ameliorate atherosclerosis in multiple mouse models [98]. Although the above studies give us some suggestions, the specific mechanisms of efferocytosis failures remain unknown and require careful assessment with *in vivo* and *in vitro* genetic causation testing in the future.

## 5. Macrophage Functional Diversity in Atherosclerosis

### 5.1. M1 and M2 Macrophages

**Figure 2:**
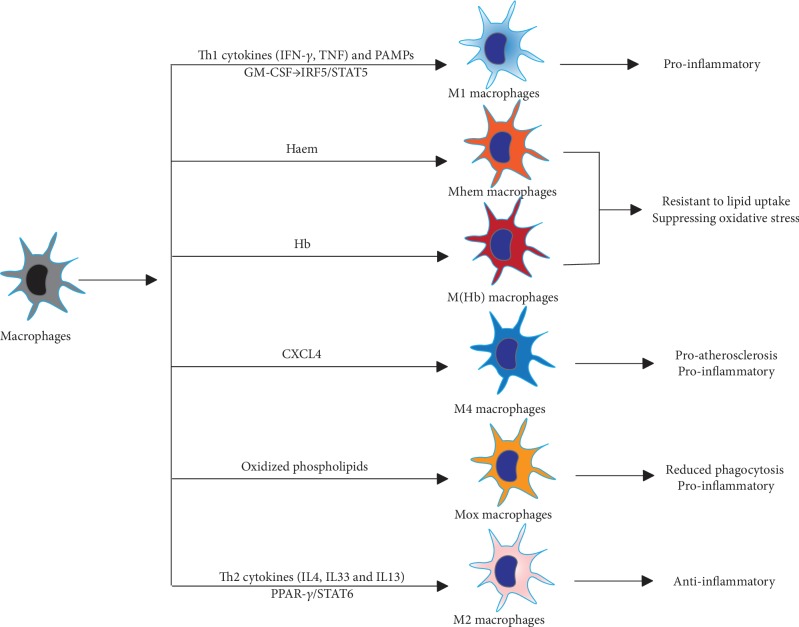
Macrophage subsets in the atherosclerotic lesion. M1 proinflammatory and M2 anti-inflammatory macrophages are polarized by Th1 and Th2 cytokines, respectively. Haem-induced phenotypes including M(Hb) and Mhem are M2-like and show anti-inflammatory effects such as resistant to lipid uptake and suppressing oxidative stress. Intermediate phenotypes Mox and M4 display reduced capacity for phagocytosis and are potentially proinflammatory by expressing proatherogenic markers. Abbreviations: CXCL4: C-X-C motif chemokine 4; GM-CSF: granulocyte macrophage colony-stimulating factor; IFN-*γ*: interferon-*γ*; IL: interleukin; IRF: interferon regulatory factor 5; PAMPs: pathogen-associated molecular complexes; STAT: signal transducer and activator of transcription; TNF: tumor necrosis factor.

As with the well-established T cell polarization system that is based on transcriptome, phenotype, and function, lesional macrophages are greatly influenced by the microenvironment signals and are polarized into different classes with diverse phenotypes and functions (Figure 2) [12]. Accurate research on macrophage differentiation and heterogeneity is limited by macrophage instability during the isolation process and phenotype differences between animal models and humans.

In the simplified dichotomy, immune-activated proinflammatory macrophages (M1) and immunomodulatory alternatively activated macrophages (M2) are the most classical classification, mirroring the two types of T helper cells (Th1 and Th2), and represent the extreme phenotypes of the complicated activation states [103]. M1 macrophages are typically polarized by Th1 cytokines, such as interferon (IFN-*γ*) and TNF and pathogen-associated molecular complexes (PAMPs), including lipopolysaccharides and lipoproteins [12]. Granulocyte macrophage colony-stimulating factor (GM-CSF) also promotes a proinflammatory M1 state through interferon regulatory factor 5 (IRF5) [104]. M1 macrophages produce high levels of proinflammatory cytokines, such as IL-6, IL-12, IL-23, TNF-*α*, and IL-1*β*, and Th1 recruitment-associated chemokines, such as CXCL-9, CXCL-10, and CXCL-11, and low levels of IL-10 [105, 106]. However, chronic M1 macrophage activation can also induce the NADPH oxidase system and subsequently generate ROS and NO, inducing chronic tissue damage and impairing wound healing [107]. At this point, M2 macrophages are necessary to counterbalance the proinflammatory response and function to modulate inflammation, scavenge apoptotic cells, accelerate angiogenesis and fibrosis, and promote tissue repair [108]. M2 macrophages are mainly induced in response to Th2-related cytokines, including IL-4, IL-33, and IL-13 [108]. Activated M2 macrophages are immunomodulatory and characterized by low levels of IL-12 and high levels of anti-inflammatory cytokines such as IL-10 and TGF-*β* and chemokines CCL17, CCL22, and CCL24 [14]. In fact, according to the differences in activation cues and gene expression profiles, M2 macrophages can be further divided into four subgroups, M2a, M2b, M2c, and M2d [14, 109]. M2a macrophages are induced by IL­4 and IL­13 and are characterized by high levels of CD206 and IL-1 receptor antagonist; M2b macrophages are an exception and are induced by immune complexes, IL­1*β* and PAMPs, and produce both proinflammatory cytokines IL-1, IL-6, and TNF-*α* and the anti-inflammatory cytokine IL-10; M2c macrophages are the most prominent anti-inflammatory subtype and are induced by IL­10, TGF­*β*, and glucocorticoids and produce IL­10, TGF­*β* and pentraxin 3 (PTX3); last, M2d macrophages are induced by TLR signals and characterized by angiogenic properties, playing a role both in plaque growth and tumor progression. Activation of the peroxisome proliferator-activated receptor *γ* (PPAR-*γ*) and signal transducer and activator of transcription 6 (STAT6) pathways is the main signal for M2 polarization [109, 110]. M1 and M2 macrophages present at different regions of the plaque: M1 macrophage marker staining is mostly confined to the shoulder of rupture-prone plaques, one of the most unstable areas within the plaque, while M2 macrophage markers are mainly present in the vascular adventitia or regions of stable plaques [111]. M1 macrophages are also more abundant in the lesions of infarction and CAD patients than M2 macrophages [112, 113].

### 5.2. Other Macrophage Phenotypes

Along with a deep understanding of the phenotypes and functions of lesional macrophages, it has been clearly proven that the M1-M2 dichotomy does not actually reflect the complicated subsets of macrophages in atherosclerosis (Figure 2) [4, 12, 111]. Stimuli vary spatiotemporally and drive malleable macrophages into a broad spectrum of activation states, rather than a stable analogous polarization, which might be the reason for the difficulty in keeping phenotypes of isolated macrophages stable. A novel way to classify macrophages is by stimulus: e.g., M(IFN-*γ*), M(IL-4), and M(IL-10). Recently, Piccolo et al. induced macrophages by dual stimulation with IFN-*γ* and IL-4, which are the inducers for M1 and M2 phenotype macrophages, respectively, and found that costimulation with two opposite stimuli drove macrophages to an intermediate state that we could call M(IFN-*γ*-IL-4) and displayed both M1- and M2-type specific gene transcriptome signatures [114]. In addition to M1-M2, oxidized phospholipids can induce macrophages to a Mox phenotype via activation of the transcription factor Nrf2 in mouse models [15]. Mox macrophages constitute approximately 30% of the total macrophages in advanced atherosclerotic lesions, and these cells express proinflammatory markers, such as IL­1*β* and cyclooxygenase 2, and display defective phagocytic and chemotactic capacities [15]. Rupture of microvessels within the lesion releases erythrocytes, which can be phagocytosed by macrophages and then induce them into M(Hb) and Mhem phenotypes [16, 17]. M(Hb) macrophages can be induced *in vitro* by hemoglobin-haptoglobin complexes and present a CD206^+^ CD163^+^ phenotype. M(Hb) macrophages have increased activity of LXR­*α*, which results in increased cholesterol efflux and reduced lipid accumulation, increased ferroportin expression, which leads to reduced intracellular iron accumulation, and increased secretion of anti-inflammatory factors such as IL-10. The Mhem phenotype is polarized by heme and is characterized by increased expression of cyclic AMP­dependent transcription factor- (ATF-) 1 and heme oxygenase 1 (HO­1) and suppressed oxidative stress or lipid accumulation, sharing similar properties with H(Hb) macrophages. Both M(Hb) and Mhem cells are hemorrhage-associated phenotypes that are generally resistant to transformation to foam cells, suppressing oxidative stress and potentially serving atheroprotective roles. C­X­C motif chemokine 4 (CXCL-4) chemokine induces M4 phenotype macrophages in human atherosclerotic plaques, which are CD163^−^ and characterized by expression of MMP-7 and the calcium-binding protein S100A8 and presentation of proinflammatory and proatherogenic properties [115]. Interestingly, M1, M2, and hemorrhage-associated phenotypes can switch between one another, while the M4 macrophage phenotype seems to be irreversible [115].

## 6. Monocyte Phenotypes in Atherosclerosis

Similar to macrophages, their precursor cells, monocytes, are also induced into distinct phenotypes in the circulation before recruitment to the artery [18]. Three subsets are reported based on the expression of CD14 and CD16: classical, nonclassical, and intermediate monocytes. Classical monocytes are CD14^++^ CD16^−^ in humans and Ly6C^++^ CCR2^+^ CX3CR1^+^ in mice [116]. Classical monocytes are the majority of total monocytes, have proinflammatory features, and differentiate into macrophages and DCs [117]. Nonclassical monocytes are CD14^+^ CD16^++^ in humans and Ly6C^−^ CCR2^−^ CX3CR1^++^ in mice, circulate longer in the blood, present more M2-like properties, and may counterbalance the classical subsets [116]. Intermediate monocytes are the remaining CD14^++^ CD16^+^ subset and account for approximately 5% of the total monocyte population. Although the intermediate phenotype is the smallest subset population, most studies find positive relationships between this subtype and CVD events and plaque thinning [118]. This may be due to their CD11c integrin expression and stronger capacity to adhere to endothelium than the other two subsets [119]. Consistent with these results, a recent study utilized a novel experimental technique, time-of-flight mass cytometry, to analyze the phenotypes human monocyte subsets in CVD lesions and found that the percentage of intermediate and nonclassical monocytes was increased in the high CVD risk group [120]. It is reasonable to assume that different monocyte subsets might differentiate into distinct macrophages and further contribute to the formation of corresponding plaques with different vulnerabilities. However, thus far, no corresponding evidence is available to validate this hypothesis.

## 7. Therapeutic Strategies Targeting Macrophage-Dependent Inflammation

### 7.1. Antiatherosclerotic Biomarker Strategies

The traditional strategy to reduce CVD risk mostly focuses on the control of blood lipids, such as traditional drug statins, and antiplatelet therapy. Lowering blood lipids results in a decrease in both apoB-LP deposition and subsequent monocyte/macrophage infiltration [121, 122]. Recently, novel targeted drugs inhibiting proprotein convertase subtilisin-kexin type 9 (PCSK9), *Evolocumab* [123] and *Alirocumab* [124, 125], emerge as an add-on therapy for lowering LDL levels, both of which could prevent LDL receptor degradation, promote LDL clearance, and further reduce the risk of CAD events. Another cholesterol modulation agent, inhibition of the cholesteryl ester transfer protein (CETP), such as anacetrapib, reduces the risk of CAD in statin-treated patients [126] by raising high-density lipoprotein (HDL) and lowering LDL level [127].

With a deep understanding of the nature of atherosclerosis as chronic inflammation and the fact that macrophages are involved throughout the entire process of atherosclerosis, including lesion initiation, progression, advanced lesion necrosis, and plaque breakdown (Figure 1), strategies to modulate the proinflammatory environment in the lesion, macrophage-related responses in particular, have emerged as promising additive therapies. Notably, in addition to the LDL downregulating effect, PCSK9 inhibitors also show anti-inflammatory effects via both LDL receptor-dependent and independent pathways [128]. PCSK9 is normally expressed on atherosclerotic cells, including monocytes/macrophages, ECs, and VSMCs, and promotes the proinflammatory environment [129–131]. In PCSK9 knockout or overexpression mouse models, inflammatory cytokines such as TNF-*α*, IL-6, and monocyte chemoattractant protein-1 (MCP-1) are negatively correlated with PCSK9 expression [129, 130, 132].

Studies *in vitro* and in mice models have explored an abundance of plausible antibodies or inhibitory molecules targeted on proatherosclerotic biomarkers, such as adhesion molecules, scavenger receptors, efferocytosis-related receptors, ER stress signaling, oxidants, and macrophage inflammation. However, most strategies are far from being translated into therapeutic drugs. Several clinical trials have been undergoing targeting critical cytokines and chemokines involved in macrophage inflammation, such as CCR2, CX3CR1, TNF-*α*, IL-1*β*, and IL-6 [133]. CCR2 blockade MLN1202 [134], IL-6 receptor antagonist tocilizumab [135] (NCT02659150), IL-1*β* inhibitor Canakinumab (NCT01327846 [136]), and IL-1 blocker anakinra [137] are all demonstrated to lower blood C-reactive protein (hsCRP) levels, which is a reliable marker of proatherogenic inflammation. Canakinumab was indicated to reduce the CAD risk with a dose-dependent feature [136], and patients treated with Canakinumab who achieved hsCRP concentrations less than 2 mg/L benefited a 25% reduction of CVD risk [138]. Treatment with TNF-*α* blockers etanercept or infliximab and methotrexate is associated with nearly 30% lower CVD risk among patients with rheumatoid arthritis [139]. Antioxidant therapy such as febuxostat, an inhibitor of xanthine oxidase, also functions partly through effects on macrophages [140]. Antioxidant therapy could modify the process of atherogenesis by preventing oxidation of LDL, formation of ROS, and subsequent release of inflammatory cytokines in macrophages [141]. Clinical trials targeting other molecules, including CX3CR1, IL-12, LXR, IRF5, and PPAR-*γ*, are still on the way and have not reached any conclusion.

Notably, macrophage-related proatherosclerotic inflammatory responses are not easily distinguished from host defense, so therapeutic measures are likely to cause increased susceptibility to infections. The local target-based delivery systems would possibly lessen this problem by improving the drug efficacy. Anti-inflammatory biomarker drugs such as antibodies and siRNA carried by nanoparticles (NPs) [142] or stents [143, 144] have been applied in animal models and *in vitro* studies to electively clear macrophage-related inflammation.

### 7.2. Strategies Targeting Macrophages

Antiatherosclerotic biomarkers or lipid modulation strategies are nonspecific measures that suppress the functions of macrophages and other cells in the plaque, such as SMCs and ECs. However, therapies directly and specifically targeting macrophages are scarce, and studies have thus far been preclinical work, possibly owing to the complicated phenotypical and functional heterogeneity of lesional macrophages.

Currently, novel drug delivery systems such as NPs, stents, liposomes, glucan shell microparticles, oligopeptide complexes, and monoclonal antibodies make it possible to selectively modify macrophages. Macrophage surface markers such as F4/80, CD11b, CD68, CD206, and scavenger receptors provide unique targets for all macrophages or different subsets [145]. Coupled with surface coating receptors or depending on their chemical properties, these systems could deliver drugs or RNAi to local atherosclerotic plaques or specific macrophage subsets and exert modifications with minimal off-target effects and toxicity [146]. Once in proximity to or inside of the macrophages, diverse approaches could be applied to modulate macrophages, including inducing cell apoptosis [147], inhibiting cell proliferation [148], and introducing anti-inflammatory agents [149]. Verheye et al. found that the rapamycin inhibitor everolimus delivered to plaques in a stent-based rabbit model led to autophagy in macrophages without affecting the number of SMCs [143]. In a high-fat diet mouse model, clodronate liposome injection effectively depleted visceral adipose tissue macrophages and blocked high-fat diet-induced weight gain and metabolic disorders [150]. Stoneman et al. explored the effect of total macrophage- and blood monocyte-targeted ablation by building a CD11b-diphtheria toxin (DT) receptor (DTR) transgenic mouse model via administration of DT [151]. Plaques were remarkedly reduced when DT was given at the initiation time of atherogenesis, while established plaques were not affected by DT, even though macrophages were reduced to a similar level, which suggests that the atherogenesis process is more sensitive to reduced monocytes/macrophages than stable plaques [50]. Unfortunately, although promising, all evidence has been developed *in vitro* or in animal models, and further studies are needed for more novel drugs and clinical translation.

In addition to the removal of macrophages, influencing macrophage polarization to an anti-inflammatory phenotype, M2 macrophages, rather than the M1 phenotype, is another option [152]. Any factor affecting M2 polarization signals might be a potential target. For example, inhibitors of dipeptidyl peptidase (DPP), such as gliptins and sitagliptin, are suggested to be able to promote M2 polarization *in vitro* via SDF-1/CXCR4 signaling [153]. Thiazolidinediones (TZDs), such as rosiglitazone and pioglitazone, activators of PPAR-*γ*, can promote monocytes to polarize to the M2 phenotype by modifying the expression of M2 markers, such as mannose receptor (MR) and CD163 [110].

## 8. Conclusions and Perspectives

Macrophages, the major immune cell population in arterial plaques, have been proven to play critical roles in the initiation and progression of atherosclerosis. Lesion-derived signals induce macrophages into complicated subsets with distinct gene expression profiles, phenotypes, and functions. Based on these results, several strategies are suggested, including blocking proinflammatory cytokines and chemokines, activating anti-inflammatory macrophages, depolarizing macrophages, and enhancing efferocytosis.

Although a number of studies have confirmed the critical functions of macrophages in atherosclerosis, many important problems remain unsolved. For example, the origins of macrophages from different organs or systems differ greatly, yet little is known about the proportions of proliferating resident macrophages or macrophages derived from circulating monocytes. This has important implications for the effectiveness of targeted drug therapy. In addition, the exact reason for the relation between intermediate monocytes and prognosis in CVD patients also needs to be clarified, as well as the functions of classical and nonclassical monocytes. In addition, more advanced techniques such as mass cytometry and single cell sequencing are needed to fully and more accurately characterize macrophage subsets and exploit novel therapeutic targets. Finally, much research is still needed before translating preclinical strategies directly targeting macrophages into clinical practice, including specific macrophage-targeted drugs and other targets, such as genetic modification.

## References

[1] Dahlof B. (2010). Cardiovascular disease risk factors: epidemiology and risk assessment. *The American Journal of Cardiology*.

[2] Moore K. J., Tabas I. (2011). Macrophages in the pathogenesis of atherosclerosis. *Cell*.

[3] Moore K. J., Sheedy F. J., Fisher E. A. (2013). Macrophages in atherosclerosis: a dynamic balance. *Nature Reviews Immunology*.

[4] Park I., Kassiteridi C., Monaco C. (2017). Functional diversity of macrophages in vascular biology and disease. *Vascular Pharmacology*.

[5] Gimbrone M. A., Garcia-Cardena G. (2016). Endothelial cell dysfunction and the pathobiology of atherosclerosis. *Circulation Research*.

[6] Robbins C. S., Hilgendorf I., Weber G. F. (2013). Local proliferation dominates lesional macrophage accumulation in atherosclerosis. *Nature Medicine*.

[7] Lusis A. J. (2000). Atherosclerosis. *Nature*.

[8] Libby P., Aikawa M., Schonbeck U. (2000). Cholesterol and atherosclerosis. *Biochimica et Biophysica Acta (BBA) - Molecular and Cell Biology of Lipids*.

[9] Virmani R., Burke A. P., Kolodgie F. D., Farb A. (2002). Vulnerable plaque: the pathology of unstable coronary lesions. *Journal of Interventional Cardiology*.

[10] Hellings W. E., Moll F. L., de Vries J. P. (2008). Atherosclerotic plaque composition and occurrence of restenosis after carotid endarterectomy. *JAMA*.

[11] Merckelbach S., Leunissen T., Vrijenhoek J., Moll F., Pasterkamp G., de Borst G. J. (2016). Clinical risk factors and plaque characteristics associated with new development of contralateral stenosis in patients undergoing carotid endarterectomy. *Cerebrovascular Diseases*.

[12] Chinetti-Gbaguidi G., Colin S., Staels B. (2015). Macrophage subsets in atherosclerosis. *Nature Reviews Cardiology*.

[13] Goerdt S., Politz O., Schledzewski K. (2000). Alternative versus classical activation of macrophages. *Pathobiology*.

[14] Mantovani A., Sica A., Sozzani S., Allavena P., Vecchi A., Locati M. (2004). The chemokine system in diverse forms of macrophage activation and polarization. *Trends in Immunology*.

[15] Kadl A., Meher A. K., Sharma P. R. (2010). Identification of a novel macrophage phenotype that develops in response to atherogenic phospholipids via Nrf2. *Circulation Research*.

[16] Finn A. V., Nakano M., Polavarapu R. (2012). Hemoglobin directs macrophage differentiation and prevents foam cell formation in human atherosclerotic plaques. *Journal of the American College of Cardiology*.

[17] Boyle J. J., Johns M., Kampfer T. (2012). Activating transcription factor 1 directs Mhem atheroprotective macrophages through coordinated iron handling and foam cell protection. *Circulation Research*.

[18] Murphy A. J., Akhtari M., Tolani S. (2011). ApoE regulates hematopoietic stem cell proliferation, monocytosis, and monocyte accumulation in atherosclerotic lesions in mice. *The Journal of Clinical Investigation*.

[19] Serbina N. V., Pamer E. G. (2006). Monocyte emigration from bone marrow during bacterial infection requires signals mediated by chemokine receptor CCR2. *Nature Immunology*.

[20] Robbins C. S., Chudnovskiy A., Rauch P. J. (2012). Extramedullary hematopoiesis generates Ly-6C^high^ monocytes that infiltrate atherosclerotic lesions. *Circulation*.

[21] van Furth R., Cohn Z. A. (1968). The origin and kinetics of mononuclear phagocytes. *Journal of Experimental Medicine*.

[22] Ginhoux F., Greter M., Leboeuf M. (2010). Fate mapping analysis reveals that adult microglia derive from primitive macrophages. *Science*.

[23] O’Koren E. G., Mathew R., Saban D. R. (2016). Fate mapping reveals that microglia and recruited monocyte-derived macrophages are definitively distinguishable by phenotype in the retina. *Scientific Reports*.

[24] Epelman S., Lavine K. J., Randolph G. J. (2014). Origin and functions of tissue macrophages. *Immunity*.

[25] Ajami B., Bennett J. L., Krieger C., Tetzlaff W., Rossi F. M. V. (2007). Local self-renewal can sustain CNS microglia maintenance and function throughout adult life. *Nature Neuroscience*.

[26] Chappell J., Harman J. L., Narasimhan V. M. (2016). Extensive proliferation of a subset of differentiated, yet plastic, medial vascular smooth muscle cells contributes to neointimal formation in mouse injury and atherosclerosis models. *Circulation Research*.

[27] Andreeva E. R., Pugach I. M., Orekhov A. N. (1997). Subendothelial smooth muscle cells of human aorta express macrophage antigen in situ and in vitro. *Atherosclerosis*.

[28] Allahverdian S., Chehroudi A. C., McManus B. M., Abraham T., Francis G. A. (2014). Contribution of intimal smooth muscle cells to cholesterol accumulation and macrophage-like cells in human atherosclerosis. *Circulation*.

[29] Shankman L. S., Gomez D., Cherepanova O. A. (2015). KLF4-dependent phenotypic modulation of smooth muscle cells has a key role in atherosclerotic plaque pathogenesis. *Nature Medicine*.

[30] Zhu P., Huang L., Ge X., Yan F., Wu R., Ao Q. (2006). Transdifferentiation of pulmonary arteriolar endothelial cells into smooth muscle‐like cells regulated by myocardin involved in hypoxia‐induced pulmonary vascular remodelling. *International Journal of Experimental Pathology*.

[31] Vengrenyuk Y., Nishi H., Long X. (2015). Cholesterol loading reprograms the microRNA-143/145–myocardin axis to convert aortic smooth muscle cells to a dysfunctional macrophage-like phenotype. *Arteriosclerosis, Thrombosis, and Vascular Biology*.

[32] Lhoták Š., Gyulay G., Cutz J.-C. (2016). Characterization of proliferating lesion-resident cells during all stages of atherosclerotic growth. *Journal of the American Heart Association*.

[33] Mestas J., Ley K. (2008). Monocyte-endothelial cell interactions in the development of atherosclerosis. *Trends in Cardiovascular Medicine*.

[34] McEver R. P., Moore K. L., Cummings R. D. (1995). Leukocyte trafficking mediated by selectin-carbohydrate interactions. *Journal of Biological Chemistry*.

[35] Elstad M. R., La Pine T. R., Cowley F. S. (1995). P-selectin regulates platelet-activating factor synthesis and phagocytosis by monocytes. *The Journal of Immunology*.

[36] Weyrich A. S., McIntyre T. M., McEver R. P., Prescott S. M., Zimmerman G. A. (1995). Monocyte tethering by P-selectin regulates monocyte chemotactic protein-1 and tumor necrosis factor-alpha secretion. Signal integration and NF-kappa B translocation. *The Journal of Clinical Investigation*.

[37] Ma Y. Q., Plow E. F., Geng J. G. (2004). P-selectin binding to P-selectin glycoprotein ligand-1 induces an intermediate state of *α*M*β*2 activation and acts cooperatively with extracellular stimuli to support maximal adhesion of human neutrophils. *Blood*.

[38] Veerman K. M., Carlow D. A., Shanina I., Priatel J. J., Horwitz M. S., Ziltener H. J. (2012). PSGL-1 regulates the migration and proliferation of CD8^+^ T cells under homeostatic conditions. *The Journal of Immunology*.

[39] Erbel C., Sato K., Meyer F. B. (2007). Functional profile of activated dendritic cells in unstable atherosclerotic plaque. *Basic Research in Cardiology*.

[40] An G., Wang H., Tang R. (2008). P-selectin glycoprotein ligand-1 is highly expressed on Ly-6C^hi^ monocytes and a major determinant for Ly-6C^hi^ monocyte recruitment to sites of atherosclerosis in mice. *Circulation*.

[41] Luo W., Wang H., Ohman M. K. (2012). P-selectin glycoprotein ligand-1 deficiency leads to cytokine resistance and protection against atherosclerosis in apolipoprotein E deficient mice. *Atherosclerosis*.

[42] Ley K., Kansas G. S. (2004). Selectins in T-cell recruitment to non-lymphoid tissues and sites of inflammation. *Nature Reviews Immunology*.

[43] Dong Z. M., Chapman S. M., Brown A. A., Frenette P. S., Hynes R. O., Wagner D. D. (1998). The combined role of P- and E-selectins in atherosclerosis. *The Journal of Clinical Investigation*.

[44] Huo Y., Ley K. (2001). Adhesion molecules and atherogenesis. *Acta Physiologica Scandinavica*.

[45] Shattil S. J., Ginsberg M. H. (1997). Perspectives series: cell adhesion in vascular biology. Integrin signaling in vascular biology. *The Journal of Clinical Investigation*.

[46] Cybulsky M. I., Iiyama K., Li H. (2001). A major role for VCAM-1, but not ICAM-1, in early atherosclerosis. *The Journal of Clinical Investigation*.

[47] Huo Y., Hafezi-Moghadam A., Ley K. (2000). Role of vascular cell adhesion molecule-1 and fibronectin connecting segment-1 in monocyte rolling and adhesion on early atherosclerotic lesions. *Circulation Research*.

[48] Ramos C. L., Huo Y., Jung U. (1999). Direct demonstration of P-selectin– and VCAM-1–dependent mononuclear cell rolling in early atherosclerotic lesions of apolipoprotein E–deficient mice. *Circulation Research*.

[49] Koenen R. R., von Hundelshausen P., Nesmelova I. V. (2009). Disrupting functional interactions between platelet chemokines inhibits atherosclerosis in hyperlipidemic mice. *Nature Medicine*.

[50] Combadière C., Potteaux S., Rodero M. (2008). Combined inhibition of CCL2, CX3CR1, and CCR5 abrogates Ly6C^hi^ and Ly6C^lo^ Monocytosis and almost abolishes atherosclerosis in hypercholesterolemic mice. *Circulation*.

[51] Veillard N. R., Steffens S., Pelli G. (2005). Differential influence of chemokine receptors CCR2 and CXCR3 in development of atherosclerosis in vivo. *Circulation*.

[52] Tacke F., Alvarez D., Kaplan T. J. (2007). Monocyte subsets differentially employ CCR2, CCR5, and CX3CR1 to accumulate within atherosclerotic plaques. *The Journal of Clinical Investigation*.

[53] Goossens P., Gijbels M. J., Zernecke A. (2010). Myeloid type I interferon signaling promotes atherosclerosis by stimulating macrophage recruitment to lesions. *Cell Metabolism*.

[54] Williams K. J., Tabas I. (1995). The response-to-retention hypothesis of early atherogenesis. *Arteriosclerosis, Thrombosis, and Vascular Biology*.

[55] Leake D. S. (1993). Oxidised low density lipoproteins and atherogenesis. *Heart*.

[56] Kunjathoor V. V., Febbraio M., Podrez E. A. (2002). Scavenger receptors class A-I/II and CD36 are the principal receptors responsible for the uptake of modified low density lipoprotein leading to lipid loading in macrophages. *Journal of Biological Chemistry*.

[57] Moore K. J., Kunjathoor V. V., Koehn S. L. (2005). Loss of receptor-mediated lipid uptake via scavenger receptor A or CD36 pathways does not ameliorate atherosclerosis in hyperlipidemic mice. *The Journal of Clinical Investigation*.

[58] Crucet M., Wüst S. J. A., Spielmann P., Lüscher T. F., Wenger R. H., Matter C. M. (2013). Hypoxia enhances lipid uptake in macrophages: role of the scavenger receptors Lox1, SRA, and CD36. *Atherosclerosis*.

[59] Lillis A. P., Muratoglu S. C., Au D. T. (2015). LDL receptor-related protein-1 (LRP1) regulates cholesterol accumulation in macrophages. *PLoS One*.

[60] Spann N. J., Garmire L. X., McDonald J. (2012). Regulated accumulation of desmosterol integrates macrophage lipid metabolism and inflammatory responses. *Cell*.

[61] Kruth H. S., Jones N. L., Huang W. (2005). Macropinocytosis is the endocytic pathway that mediates macrophage foam cell formation with native low density lipoprotein. *Journal of Biological Chemistry*.

[62] Zhu G., Xu Y., Cen X., Nandakumar K. S., Liu S., Cheng K. (2018). Targeting pattern-recognition receptors to discover new small molecule immune modulators. *European Journal of Medicinal Chemistry*.

[63] Stewart C. R., Stuart L. M., Wilkinson K. (2010). CD36 ligands promote sterile inflammation through assembly of a Toll-like receptor 4 and 6 heterodimer. *Nature Immunology*.

[64] Mullick A. E., Tobias P. S., Curtiss L. K. (2005). Modulation of atherosclerosis in mice by Toll-like receptor 2. *The Journal of Clinical Investigation*.

[65] Björkbacka H., Kunjathoor V. V., Moore K. J. (2004). Reduced atherosclerosis in MyD88-null mice links elevated serum cholesterol levels to activation of innate immunity signaling pathways. *Nature Medicine*.

[66] Michelsen K. S., Wong M. H., Shah P. K. (2004). Lack of Toll-like receptor 4 or myeloid differentiation factor 88 reduces atherosclerosis and alters plaque phenotype in mice deficient in apolipoprotein E. *Proceedings of the National Academy of Sciences of the United States of America*.

[67] Gareus R., Kotsaki E., Xanthoulea S. (2008). Endothelial Cell-Specific NF-*κ*B Inhibition Protects Mice from Atherosclerosis. *Cell Metabolism*.

[68] Duewell P., Kono H., Rayner K. J. (2010). NLRP3 inflammasomes are required for atherogenesis and activated by cholesterol crystals. *Nature*.

[69] Zheng F., Xing S., Gong Z., Mu W., Xing Q. (2014). Silence of NLRP3 suppresses atherosclerosis and stabilizes plaques in apolipoprotein E-deficient mice. *Mediators of Inflammation*.

[70] Schonbeck U., Libby P. (2001). The CD40/CD154 receptor/ligand dyad. *Cellular and Molecular Life Sciences*.

[71] Anand S. X., Viles-Gonzalez J. F., Badimon J. J., Cavusoglu E., Marmur J. D. (2003). Membrane-associated CD40L and sCD40L in atherothrombotic disease. *Thrombosis and Haemostasis*.

[72] Chen Y., Chen J., Xiong Y. (2006). Internalization of CD40 regulates its signal transduction in vascular endothelial cells. *Biochemical and Biophysical Research Communications*.

[73] Lessner S. M., Martinson D. E., Galis Z. S. (2004). Compensatory vascular remodeling during atherosclerotic lesion growth depends on matrix metalloproteinase-9 activity. *Arteriosclerosis, Thrombosis, and Vascular Biology*.

[74] Ivan E., Khatri J. J., Johnson C. (2002). Expansive arterial remodeling is associated with increased neointimal macrophage foam cell content: the murine model of macrophage-rich carotid artery lesions. *Circulation*.

[75] Boyle J. J., Weissberg P. L., Bennett M. R. (2002). Human macrophage-induced vascular smooth muscle cell apoptosis requires NO enhancement of Fas/Fas-L interactions. *Arteriosclerosis, Thrombosis, and Vascular Biology*.

[76] Boyle J. J., Bowyer D. E., Weissberg P. L., Bennett M. R. (2001). Human blood-derived macrophages induce apoptosis in human plaque-derived vascular smooth muscle cells by Fas-ligand/Fas interactions. *Arteriosclerosis, Thrombosis, and Vascular Biology*.

[77] Boyle J. J., Weissberg P. L., Bennett M. R. (2003). Tumor necrosis Factor-*α* promotes macrophage-induced vascular smooth muscle cell apoptosis by direct and autocrine mechanisms. *Arteriosclerosis, Thrombosis, and Vascular Biology*.

[78] Fadok V. A., Bratton D. L., Konowal A., Freed P. W., Westcott J. Y., Henson P. M. (1998). Macrophages that have ingested apoptotic cells in vitro inhibit proinflammatory cytokine production through autocrine/paracrine mechanisms involving TGF-beta, PGE2, and PAF. *The Journal of Clinical Investigation*.

[79] LeBaron R. G., Bezverkov K. I., Zimber M. P., Pavelec R., Skonier J., Purchio A. F. (1995). *β*IG-H3, a Novel Secretory Protein Inducible by Transforming Growth Factor-*β*, Is Present in Normal Skin and Promotes the Adhesion and Spreading of Dermal Fibroblasts In Vitro. *The Journal of Investigative Dermatology*.

[80] Chistiakov D. A., Sobenin I. A., Orekhov A. N. (2013). Vascular extracellular matrix in atherosclerosis. *Cardiology in Review*.

[81] Newby A. C. (2005). Dual role of matrix metalloproteinases (matrixins) in intimal thickening and atherosclerotic plaque rupture. *Physiological Reviews*.

[82] Gough P. J., Gomez I. G., Wille P. T., Raines E. W. (2006). Macrophage expression of active MMP-9 induces acute plaque disruption in apoE-deficient mice. *The Journal of Clinical Investigation*.

[83] Johnson J., George S., Newby A., Jackson C. (2003). 3HT03-3 matrix metalloproteinases-9 and -12 have opposite effects on atherosclerotic plaque stability. *Atherosclerosis Supplements*.

[84] Tabas I. (2010). Macrophage death and defective inflammation resolution in atherosclerosis. *Nature Reviews Immunology*.

[85] Zhang C., Syed T. W., Liu R., Yu J. (2017). Role of endoplasmic reticulum stress, autophagy, and inflammation in cardiovascular disease. *Frontiers in Cardiovascular Medicine*.

[86] Gotoh T., Endo M., Oike Y. (2011). Endoplasmic reticulum stress-related inflammation and cardiovascular diseases. *International Journal of Inflammation*.

[87] Bessone F., Razori M. V., Roma M. G. (2019). Molecular pathways of nonalcoholic fatty liver disease development and progression. *Cellular and Molecular Life Sciences*.

[88] Tabas I., Tall A., Accili D. (2010). The impact of macrophage insulin resistance on advanced atherosclerotic plaque progression. *Circulation Research*.

[89] Myoishi M., Hao H., Minamino T. (2007). Increased endoplasmic reticulum stress in atherosclerotic plaques associated with acute coronary syndrome. *Circulation*.

[90] Tsukano H., Gotoh T., Endo M. (2010). The endoplasmic reticulum stress-C/EBP homologous protein pathway-mediated apoptosis in macrophages contributes to the instability of atherosclerotic plaques. *Arteriosclerosis, Thrombosis, and Vascular Biology*.

[91] Thorp E., Li G., Seimon T. A., Kuriakose G., Ron D., Tabas I. (2009). Reduced apoptosis and plaque necrosis in advanced atherosclerotic lesions of Apoe−/− and Ldlr−/− mice lacking CHOP. *Cell Metabolism*.

[92] Deng J., Lu P. D., Zhang Y. (2004). Translational repression mediates activation of nuclear factor kappa B by phosphorylated translation initiation factor 2. *Molecular and Cellular Biology*.

[93] Hu P., Han Z., Couvillon A. D., Kaufman R. J., Exton J. H. (2006). Autocrine tumor necrosis factor alpha links endoplasmic reticulum stress to the membrane death receptor pathway through IRE1*α*-mediated NF-*κ*B activation and down-regulation of TRAF2 expression. *Molecular and Cellular Biology*.

[94] Cullinan S. B., Diehl J. A. (2006). Coordination of ER and oxidative stress signaling: the PERK/Nrf2 signaling pathway. *The International Journal of Biochemistry & Cell Biology*.

[95] Gotoh T., Mori M. (2006). Nitric oxide and endoplasmic reticulum stress. *Arteriosclerosis, Thrombosis, and Vascular Biology*.

[96] Navarro-Yepes J., Burns M., Anandhan A. (2014). Oxidative stress, redox signaling, and autophagy: cell death versus survival. *Antioxidants & Redox Signaling*.

[97] Schrijvers D. M., de Meyer G. R. Y., Kockx M. M., Herman A. G., Martinet W. (2005). Phagocytosis of apoptotic cells by macrophages is impaired in atherosclerosis. *Arteriosclerosis, Thrombosis, and Vascular Biology*.

[98] Kojima Y., Volkmer J. P., McKenna K. (2016). CD47-blocking antibodies restore phagocytosis and prevent atherosclerosis. *Nature*.

[99] Kojima Y., Downing K., Kundu R. (2014). Cyclin-dependent kinase inhibitor 2B regulates efferocytosis and atherosclerosis. *The Journal of Clinical Investigation*.

[100] Gillotte-Taylor K., Boullier A., Witztum J. L., Steinberg D., Quehenberger O. (2001). Scavenger receptor class B type I as a receptor for oxidized low density lipoprotein. *Journal of Lipid Research*.

[101] Yvan-Charvet L., Pagler T. A., Seimon T. A. (2010). ABCA1 and ABCG1 protect against oxidative stress–induced macrophage apoptosis during efferocytosis. *Circulation Research*.

[102] Cai B., Thorp E. B., Doran A. C. (2017). MerTK receptor cleavage promotes plaque necrosis and defective resolution in atherosclerosis. *The Journal of Clinical Investigation*.

[103] Mills C. D., Kincaid K., Alt J. M., Heilman M. J., Hill A. M. (2000). M-1/M-2 macrophages and the Th1/Th2 paradigm. *The Journal of Immunology*.

[104] Krausgruber T., Blazek K., Smallie T. (2011). IRF5 promotes inflammatory macrophage polarization and T_H_1-T_H_17 responses. *Nature Immunology*.

[105] Verreck F. A. W., de Boer T., Langenberg D. M. L. (2004). Human IL-23-producing type 1 macrophages promote but IL-10-producing type 2 macrophages subvert immunity to (myco)bacteria. *Proceedings of the National Academy of Sciences of the United States of America*.

[106] Mosser D. M. (2003). The many faces of macrophage activation. *Journal of Leukocyte Biology*.

[107] Kotwal G. J., Chien S. (2017). Macrophage differentiation in normal and accelerated wound healing. *Results and Problems in Cell Differentiation*.

[108] Gordon S., Martinez F. O. (2010). Alternative activation of macrophages: mechanism and functions. *Immunity*.

[109] Juhas U., Ryba-Stanislawowska M., Szargiej P., Mysliwska J. (2015). Different pathways of macrophage activation and polarization. *Postȩpy Higieny i Medycyny Doświadczalnej*.

[110] Bouhlel M. A., Derudas B., Rigamonti E. (2007). PPAR*γ* activation primes human monocytes into alternative M2 macrophages with anti-inflammatory properties. *Cell Metabolism*.

[111] Stöger J. L., Gijbels M. J. J., van der Velden S. (2012). Distribution of macrophage polarization markers in human atherosclerosis. *Atherosclerosis*.

[112] Hirata Y., Tabata M., Kurobe H. (2011). Coronary atherosclerosis is associated with macrophage polarization in epicardial adipose tissue. *Journal of the American College of Cardiology*.

[113] de Gaetano M., Crean D., Barry M., Belton O. (2016). M1- and M2-type macrophage responses are predictive of adverse outcomes in human atherosclerosis. *Frontiers in Immunology*.

[114] Piccolo V., Curina A., Genua M. (2017). Opposing macrophage polarization programs show extensive epigenomic and transcriptional cross-talk. *Nature Immunology*.

[115] Gleissner C. A., Shaked I., Little K. M., Ley K. (2010). CXC chemokine ligand 4 induces a unique transcriptome in monocyte-derived macrophages. *The Journal of Immunology*.

[116] Geissmann F., Jung S., Littman D. R. (2003). Blood monocytes consist of two principal subsets with distinct migratory properties. *Immunity*.

[117] Ghattas A., Griffiths H. R., Devitt A., Lip G. Y., Shantsila E. (2013). Monocytes in coronary artery disease and atherosclerosis: where are we now?. *Journal of the American College of Cardiology*.

[118] Biessen E. A. L., Wouters K. (2017). Macrophage complexity in human atherosclerosis: opportunities for treatment?. *Current Opinion in Lipidology*.

[119] Foster G. A., Gower R. M., Stanhope K. L., Havel P. J., Simon S. I., Armstrong E. J. (2013). On-chip phenotypic analysis of inflammatory monocytes in atherogenesis and myocardial infarction. *Proceedings of the National Academy of Sciences of the United States of America*.

[120] Merino A., Buendia P., Martin-Malo A., Aljama P., Ramirez R., Carracedo J. (2011). Senescent CD14^+^CD16^+^ monocytes exhibit proinflammatory and proatherosclerotic activity. *The Journal of Immunology*.

[121] Potteaux S., Gautier E. L., Hutchison S. B. (2011). Suppressed monocyte recruitment drives macrophage removal from atherosclerotic plaques of *Apoe*^–/–^ mice during disease regression. *The Journal of Clinical Investigation*.

[122] Feig J. E., Pineda-Torra I., Sanson M. (2010). LXR promotes the maximal egress of monocyte-derived cells from mouse aortic plaques during atherosclerosis regression. *The Journal of Clinical Investigation*.

[123] Hadjiphilippou S., Ray K. K. (2017). Evolocumab and clinical outcomes in patients with cardiovascular disease. *The Journal of the Royal College of Physicians of Edinburgh*.

[124] Schwartz G. G., Steg P. G., Szarek M. (2018). Alirocumab and cardiovascular outcomes after acute coronary syndrome. *The New England Journal of Medicine*.

[125] Ray K. K., Colhoun H. M., Szarek M. (2019). Effects of alirocumab on cardiovascular and metabolic outcomes after acute coronary syndrome in patients with or without diabetes: a prespecified analysis of the ODYSSEY OUTCOMES randomised controlled trial. *The Lancet Diabetes and Endocrinology*.

[126] The HPS3/TIMI55–REVEAL Collaborative Group (2017). Effects of anacetrapib in patients with atherosclerotic vascular disease. *The New England Journal of Medicine*.

[127] Thompson A., Di Angelantonio E., Sarwar N. (2008). Association of cholesteryl ester transfer protein genotypes with CETP mass and activity, lipid levels, and coronary risk. *JAMA*.

[128] Tang Z. H., Li T. H., Peng J. (2019). PCSK9: a novel inflammation modulator in atherosclerosis?. *Journal of Cellular Physiology*.

[129] Ding Z., Liu S., Wang X. (2015). Hemodynamic shear stress via ROS modulates PCSK9 expression in human vascular endothelial and smooth muscle cells and along the mouse aorta. *Antioxidants & Redox Signaling*.

[130] Giunzioni I., Tavori H., Covarrubias R. (2016). Local effects of human PCSK9 on the atherosclerotic lesion. *The Journal of Pathology*.

[131] Bernelot Moens S. J., Neele A. E., Kroon J. (2017). PCSK9 monoclonal antibodies reverse the pro-inflammatory profile of monocytes in familial hypercholesterolaemia. *European Heart Journal*.

[132] Walley K. R., Thain K. R., Russell J. A. (2014). PCSK9 is a critical regulator of the innate immune response and septic shock outcome. *Science Translational Medicine*.

[133] Back M., Hansson G. K. (2015). Anti-inflammatory therapies for atherosclerosis. *Nature Reviews Cardiology*.

[134] Gilbert J., Lekstrom-Himes J., Donaldson D. (2011). Effect of CC chemokine receptor 2 CCR2 blockade on serum C-reactive protein in individuals at atherosclerotic risk and with a single nucleotide polymorphism of the monocyte chemoattractant protein-1 promoter region. *The American Journal of Cardiology*.

[135] Kleveland O., Kunszt G., Bratlie M. (2016). Effect of a single dose of the interleukin-6 receptor antagonist tocilizumab on inflammation and troponin T release in patients with non-ST-elevation myocardial infarction: a double-blind, randomized, placebo-controlled phase 2 trial. *European Heart Journal*.

[136] Ridker P. M., Everett B. M., Thuren T. (2017). Antiinflammatory therapy with canakinumab for atherosclerotic disease. *The New England Journal of Medicine*.

[137] Abbate A., van Tassell B. W., Biondi-Zoccai G. G. L. (2012). Blocking interleukin-1 as a novel therapeutic strategy for secondary prevention of cardiovascular events. *BioDrugs*.

[138] Ridker P. M., MacFadyen J., Everett B. M. (2018). Relationship of C-reactive protein reduction to cardiovascular event reduction following treatment with canakinumab: a secondary analysis from the CANTOS randomised controlled trial. *The Lancet*.

[139] Roubille C., Richer V., Starnino T. (2015). The effects of tumour necrosis factor inhibitors, methotrexate, non-steroidal anti-inflammatory drugs and corticosteroids on cardiovascular events in rheumatoid arthritis, psoriasis and psoriatic arthritis: a systematic review and meta-analysis. *Annals of the Rheumatic Diseases*.

[140] Nomura J., Busso N., Ives A. (2014). Xanthine oxidase inhibition by febuxostat attenuates experimental atherosclerosis in mice. *Scientific Reports*.

[141] Toledo-Ibelles P., Mas-Oliva J. (2018). Antioxidants in the fight against atherosclerosis: is this a dead end?. *Current Atherosclerosis Reports*.

[142] Kelley W. J., Safari H., Lopez-Cazares G., Eniola-Adefeso O. (2016). Vascular‐targeted nanocarriers: design considerations and strategies for successful treatment of atherosclerosis and other vascular diseases. *Wiley Interdisciplinary Reviews: Nanomedicine and Nanobiotechnology*.

[143] Verheye S., Martinet W., Kockx M. M. (2007). Selective clearance of macrophages in atherosclerotic plaques by autophagy. *Journal of the American College of Cardiology*.

[144] Nakano K., Egashira K., Ohtani K. (2007). Catheter-based adenovirus-mediated anti-monocyte chemoattractant gene therapy attenuates in-stent neointima formation in cynomolgus monkeys. *Atherosclerosis*.

[145] Gordon S., Pluddemann A., Martinez Estrada F. (2014). Macrophage heterogeneity in tissues: phenotypic diversity and functions. *Immunological Reviews*.

[146] Peterson K. R., Cottam M. A., Kennedy A. J., Hasty A. H. (2018). Macrophage-targeted therapeutics for metabolic disease. *Trends in Pharmacological Sciences*.

[147] Tang J., Lobatto M. E., Hassing L. (2015). Inhibiting macrophage proliferation suppresses atherosclerotic plaque inflammation. *Science Advances*.

[148] van Rooijen N., van Kesteren-Hendrikx E. (2003). "In Vivo" Depletion of Macrophages by Liposome-Mediated "Suicide". *Methods in Enzymology*.

[149] Dinarello C. A. (2010). Anti-inflammatory agents: present and future. *Cell*.

[150] Bu L., Gao M., Qu S., Liu D. (2013). Intraperitoneal injection of clodronate liposomes eliminates visceral adipose macrophages and blocks high-fat diet-induced weight gain and development of insulin resistance. *The AAPS Journal*.

[151] Stoneman V., Braganza D., Figg N. (2007). Monocyte/macrophage suppression in CD11b diphtheria toxin receptor transgenic mice differentially affects atherogenesis and established plaques. *Circulation Research*.

[152] Bi Y., Chen J., Hu F., Liu J., Li M., Zhao L. (2019). M2 macrophages as a potential target for antiatherosclerosis treatment. *Neural Plasticity*.

[153] Brenner C., Franz W. M., Kühlenthal S. (2015). DPP-4 inhibition ameliorates atherosclerosis by priming monocytes into M2 macrophages. *International Journal of Cardiology*.

